# Effects of an antimicrobial stewardship intervention on perioperative antibiotic prophylaxis in pediatrics

**DOI:** 10.1186/s13756-019-0464-z

**Published:** 2019-01-15

**Authors:** Daniele Donà, Dora Luise, Enrico La Pergola, Genni Montemezzo, Annachiara Frigo, Rebecca Lundin, Theoklis Zaoutis, Piergiorgio Gamba, Carlo Giaquinto

**Affiliations:** 1 Division of Pediatric Infectious Diseases, Department for Woman and Child Health, University of Padua, Padua, Italy; 2 PENTA Foundation, Padua, Italy; 3 Infectious and Tropical Diseases Department, University Hospital of Verona, Verona, Italy; 4 Pediatric Surgery Department, Department for Woman and Child Health, University of Padua, Padua, Italy; 5 Department for Cardio-Thoracic and Vascular Sciences and Public Health, University of Padua, Padua, Italy; 6 Division of Infectious Diseases, The Children’s Hospital of Philadelphia, Philadelphia, USA; 7 Padova, Italy

**Keywords:** Perioperative antimicrobial prophylaxis, Antimicrobial stewardship, Clinical pathway, Pediatric surgery

## Abstract

**Purpose:**

This study aims to determine the effectiveness of an Antimicrobial Stewardship Program based on a Clinical Pathway (CP) to improve appropriateness in perioperative antibiotic prophylaxis (PAP).

**Materials and methods:**

This pre-post quasi-experimental study was conducted in a 12 month period (six months before and six months after CP implementation), in a tertiary Pediatric Surgical Centre. All patients from 1 month to 15 years of age receiving one or more surgical procedures were eligible for inclusion. PAP was defined appropriate according to clinical practice guidelines.

**Results:**

Seven hundred sixty-six children were included in the study, 394 in pre-intervention and 372 in post-intervention. After CP implementation, there was an increase in appropriate PAP administration, as well as in the selection of the appropriate antibiotic for prophylaxis, both for monotherapy (p = 0.02) and combination therapy (p = 0.004). Even the duration of prophylaxis decreased during the post-intervention period, with an increase of correct PAP discontinuation from 45.1 to 66.7% (p < 0.001). Despite the greater use of narrow-spectrum antibiotic for fewer days, there was no increase in treatment failures (10/394 (2.5%) pre vs 7/372 (1.9%) post, p = 0.54).

**Conclusions:**

CPs can be a useful tool to improve the choice of antibiotic and the duration of PAP in pediatric patients.

## Background

Surgical Site Infection (SSI) is the second most common healthcare-associated infection [1] and Centres for Disease Control and Prevention (CDC) showed that it complicates approximately 5% [2] of surgical operations each year.

SSIs account for more than 16% [3] in adults and 17–18% [4, 5] in children of all hospital-acquired infections recorded in the National Healthcare Surveillance Safety Network of the Centres for Disease Control and Prevention (CDC) and 38% of nosocomial infections in surgical patients [2].

So far, only four studies focused on antimicrobial stewardship projects (ASP) for perioperative prophylaxis in children. Three of these studies showed an improvement of antimicrobial prescriptions after the implementation of perioperative guidelines [3, 6, 7], while Putnam et al. reported no improvement despite multiple interventions, such as creation of a targeted preincisional checklist and of a computerized order entry module [8]. These few data limit the conclusions that can be drawn about efficacy and safety of these strategies and represents important space for improvement for ASP in pediatric surgical prophylaxis on both side of Atlantic [3, 6–8].

The aim of this study is to determine the effectiveness of an ASP based on a Clinical Pathway (CP) to improve the adherence to perioperative antibiotic prophylaxis (PAP) guidelines [9] in a Pediatric Surgical Centre. A secondary aim is to evaluate the effect CP implementation on SSIs. To our knowledge, no specific guidelines on antimicrobial prophylaxis in pediatric surgery have been published so far, hence our CP has been developed according to the main guidelines for adult patients, that were published jointly by the American Society of Health-System Pharmacists (ASHP), the Infectious Diseases Society of America (IDSA), the Surgical Infection Society (SIS), and the Society for Healthcare Epidemiology of America (SHEA) in 2013 [9].

## Materials and methods

### Study design

This is a pre-post quasi-experimental study to assess the changes in PAP appropriateness during a 6-month period preceding CP implementation (per-intervention, from 1 February 2016 to 31 July 2016) and during the six months after CP implementation (post intervention, from 1 February 2017 to 31 July 2017).

The study was set at the Surgical Paediatric Unit of the Department for Women and Children Health at Padua University Hospital.

### Clinical pathway

**Fig. 1:**
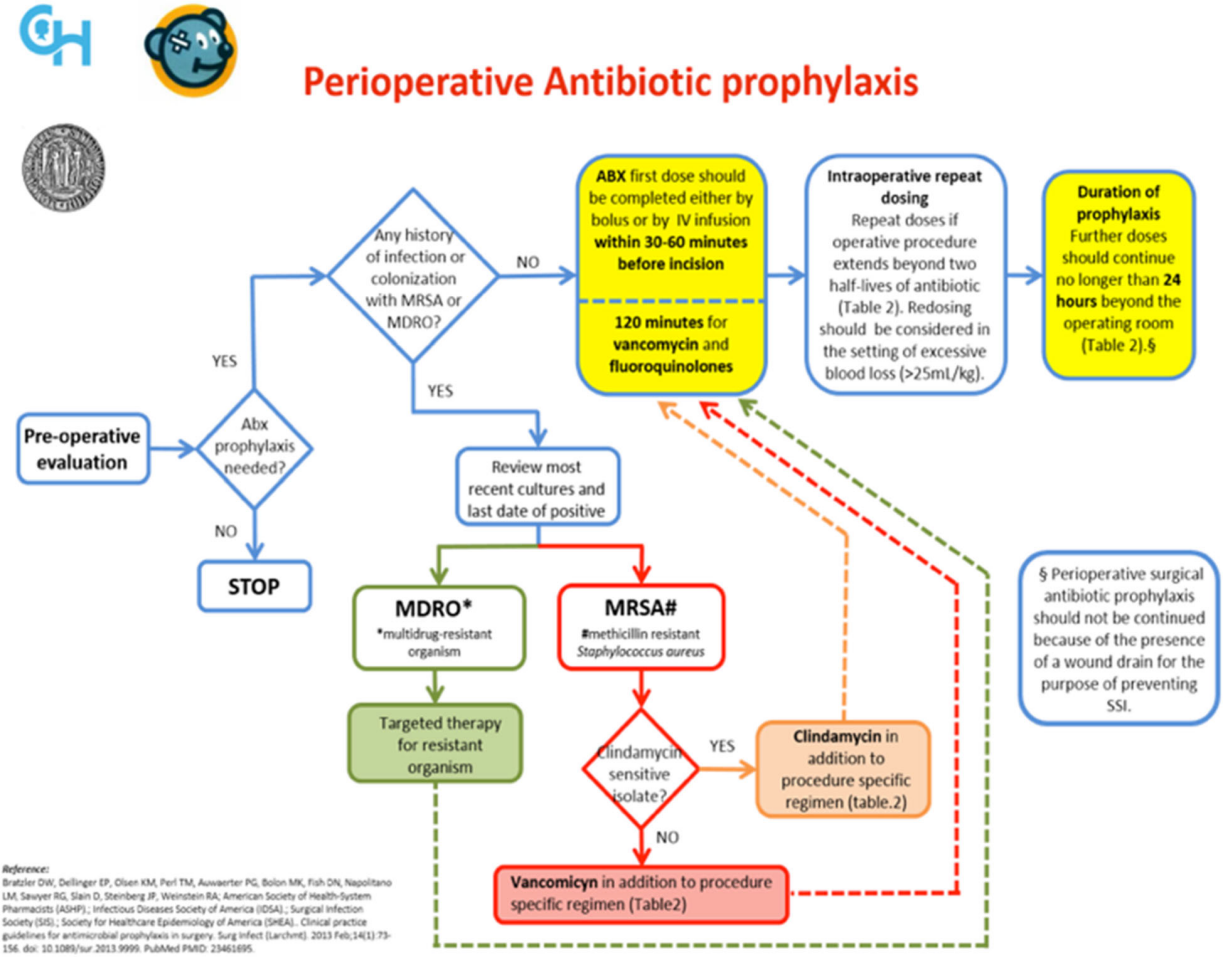
Perioperative Antibiotic Prophylaxis CP. These figures were included both in the lecture slides and in the pocket card that was delivered to all the medical staff of the Pediatric Surgery Unit. They include all the steps needed to administer a correct PAP

**Fig. 2:**
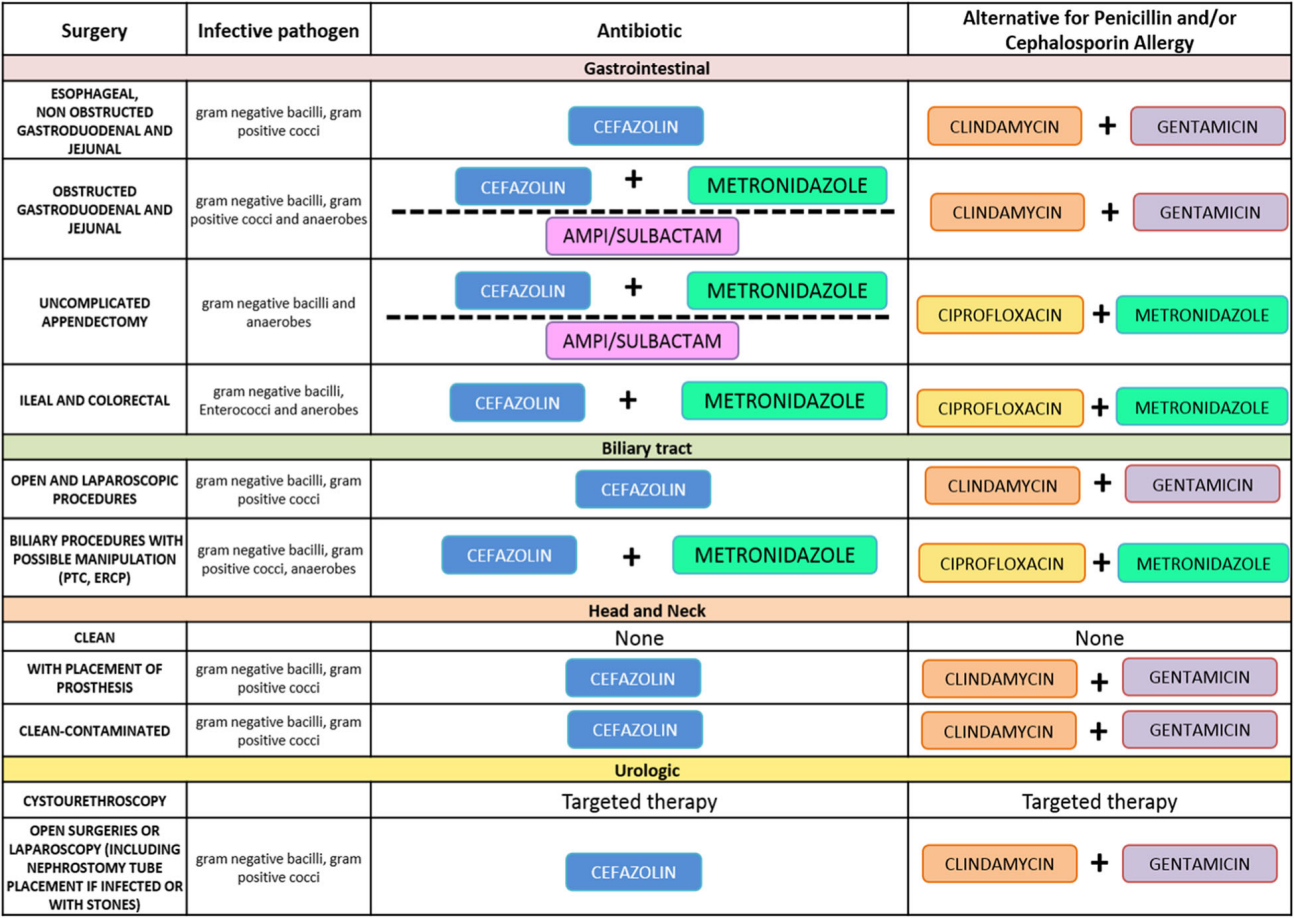
Perioperative Antibiotic Prophylaxis CP

**Fig. 3:**
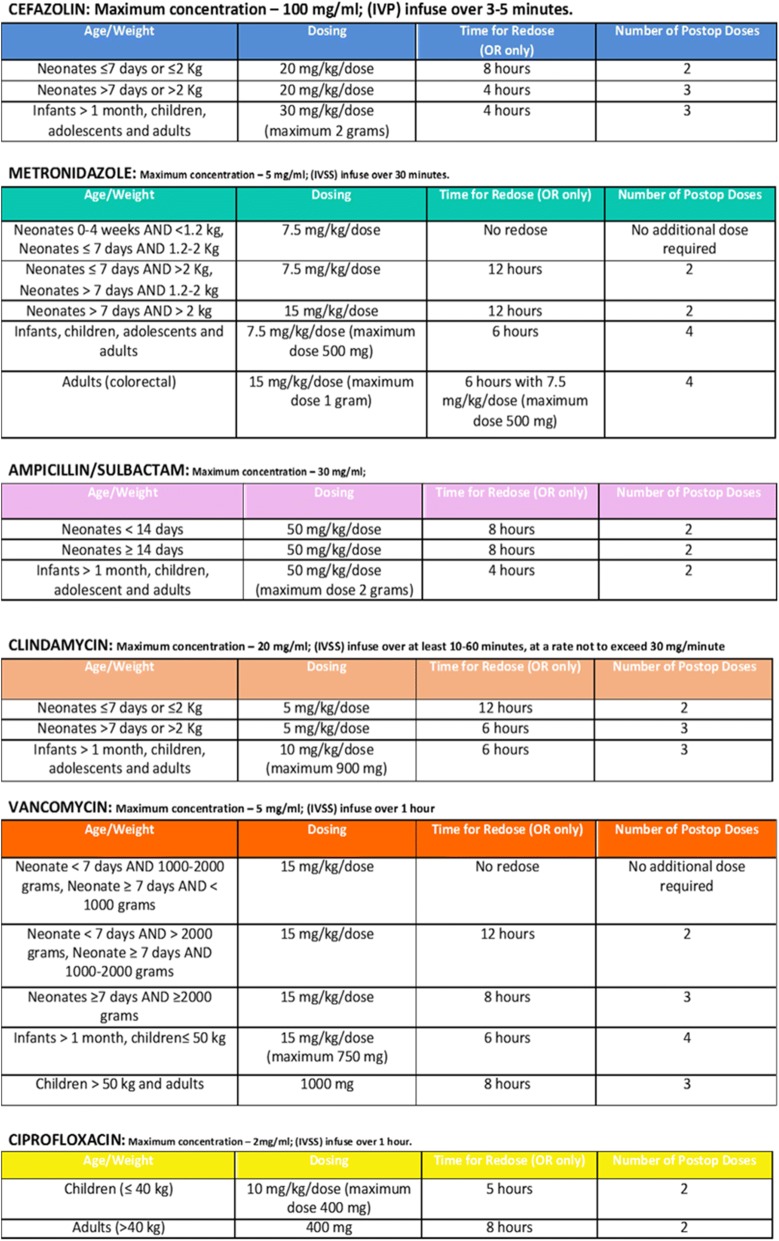
Perioperative Antibiotic Prophylaxis CP

**Fig. 4:**
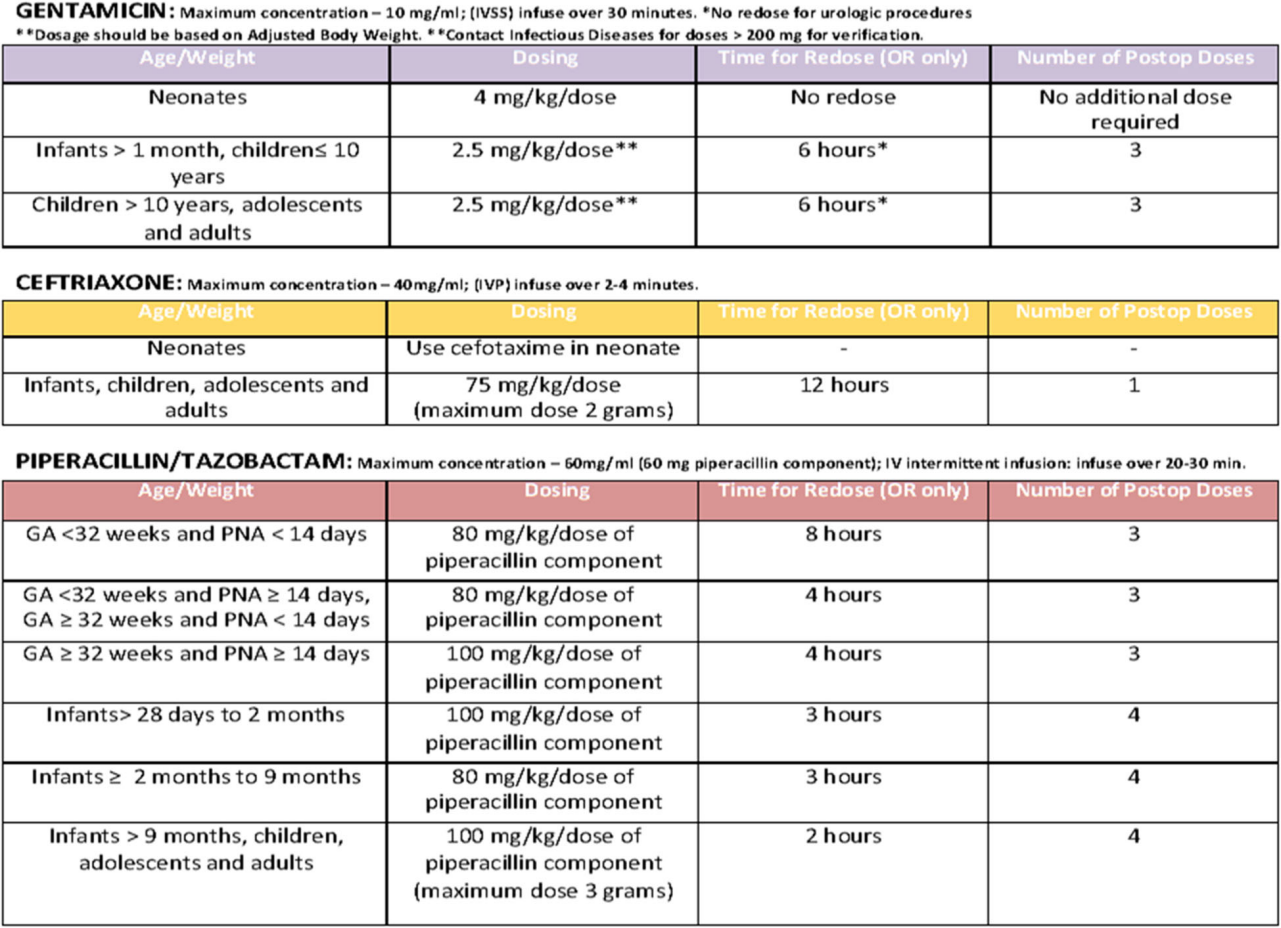
Perioperative Antibiotic Prophylaxis CP

The clinical pathway was developed by a multidisciplinary group (paediatric infectious disease, microbiology and paediatric surgery) based on the most important international clinical guidelines [9], considering our local microbiology data, and with the supervision of the paediatric infectious diseases team of Philadelphia Children’s Hospital (Figs. 1, 2, 3, and 4).

The CP details all the steps needed to administer a correct PAP.

The first step is to consider the surgical procedure (type, site and risk for developing SSIs), and consequently to decide whether to give PAP to the patient. The second step is to consider the patient’s medical history of colonization by multi-drug resistant organisms (MDROs). If the medical history is negative for MDRO, an empiric antibiotic regimen should be administered according to the type of surgical procedure. Otherwise, the prophylaxis will be targeted to the specific MDRO. Dose and duration of administration must follow the indications detailed in the CP.

The drug of choice for all surgical interventions is a first-generation cephalosporin alone. The association with metronidazole is recommended for surgical procedure with high risk for anaerobic bacteria contamination. Other molecules as clindamycin, gentamicin and ciprofloxacin should be given only to patients with proven allergy to beta-lactams antibiotic. Antibiotic first dose should be administered within 30–60 min before incision, with the exception of vancomycin and ciprofloxacin, that should be given 120 min before the incision, due to their longer half-life. An intraoperative re-dosing should be performed if the procedure extends beyond two half-lives of the antibiotic and it should be considered in the setting of excessive blood loss (> 25 mL/kg). The PAP should be discontinued within 24 h after the end of the procedure, and should not be extended longer in presence of wound drains or prosthetic implants, according to the work of Wilson and colleagues [10].

Specific recommendations for antibiotic dosages are included in the CP.

### Intervention

On 31 January 2017 the CP for PAP was implemented.

On the same day, an educational lecture was presented to all the medical staff of the Pediatric Surgery Unit. This meeting provided a review of the clinical guidelines for PAP and the potential benefits of a correct PAP, discussed the rationale for the guideline recommendations and highlighted situations where local practice in the Pediatric Surgery Unit diverged from guideline recommendations.

Following the lecture, a pocket card containing the CP was delivered to all participants and, on the same day, to all other physicians and residents who were unable to attend the seminar.

### Study population

All patients aged between one month and 15 years subjected to one or more surgical procedures were eligible to be included in our study.

Exclusion criteria were: concomitant infections, ongoing antibiotic therapy, complicated abdominal infection, immunodeficiency, immunosuppressive therapy, patients who underwent neurosurgical, vascular, ORL, and ocular procedures.

### Data source

All clinical, demographic, diagnostic and antimicrobial data were manually collected from electronic (Galileo system) or paper medical records. We used a password-protected REDCap® data collection form and we stored them in the secure server at the University of Padua. Surgical procedures were recorded using the international classification of disease, 9th revision and clinical modification (ICD 9 CM).

For every patient were recorded:

1. preoperative data including gender, age, weight;
2. procedure data including type of procedure (divided for major categories, according to the ICD-9-CM), wound class (divided in Clean, Clean-Contaminated, Contaminated and Dirty/Infected, according to the CDC’s classification [11]), duration of surgical procedure, urgency of procedure and length of hospital stay;
3. perioperative PAP data such as indication for PAP, administration of PAP, and, among those who received PAP, correctness of PAP (both agent and duration), correctness of antimicrobial agent, correctness of time of antibiotic discontinuing.
4. postprocedure data including date of medical evaluation for SSI.

PAP was defined appropriated only if the correct antimicrobial agent for the specific surgical procedures performed had been discontinued within 24 h after completion of surgery, according to clinical practice guidelines for antimicrobial prophylaxis in surgery [9].

To evaluate the effectiveness and safety of the intervention, medical records follow-up was performed to assess for SSIs within 3 months after discharge.

Privacy was guaranteed in two ways: a unique, study-specific survey number was assigned to each patient and no personally identifying data were collected.

This study was approved by the Research Ethics Committee of Department for Woman and Child Health at the University of Padua.

### Data analysis

The data were analyzed with SAS 9.4 program (SAS Institute Inc., Cary, NC, USA) for Windows.

Patient’s demographic and clinical data were analyzed in a descriptive way.

Association between the two periods was performed with Chi-square test or Fisher test for qualitative variables, and with Rank-sum Wilcoxon test for quantitative variables.

We conducted stratified analyses to assess if the effectiveness of intervention was affected by the surgical characteristics such as type of procedure, urgent surgical procedure, and duration of hospital stay. Statistical significance was considered with p < 0.05.

## Results

During the study period, 842 children underwent surgery. Of 430 children in pre-intervention period, 11 were excluded because admitted to an intensive care ward (PICU/NICU), 18 for a complicated abdominal infection and 7 for an ongoing infectious process. For post-intervention period population, 13 were excluded because admitted in the PICU/NICU, 13 for a complicated abdominal infection and 13 for an ongoing infectious process. Indeed, 766 children were included in the study, 394 in pre-intervention period and 372 in post-intervention period.

The two populations were similar in terms of sex and age, with an overall female predominance.

Baseline patient and procedure characteristics in pre- and post-intervention periods are displayed in Table 1.

**Table 1:** Patients’ main characteristics (gender, age, weight) and preoperative data (wound class, type of procedure) pre- and post-intervention periods

Patient’s characteristics	Pre-intervention (n = 394)	Post-intervention (n = 372)
Gender		
Male	122 (31%)	111 (29%)
Female	272 (69%)	267 (70%)
Median age (min-max)	5 (0–17)	5 (0–17)
Body weight (kg)	20 (2.3–74)	19 (2.1–72)
Wound class		
Clean (C)	300 (76.1%)	301 (80.9%)
Clean contaminated (CC)	63 (16%)	52 (14%)
Contaminated (CO)	31 (7.9%)	19 (5.1%)
Dirty-infected (D)	0 (0%)	0 (0%)
Type of procedure		
Appendectomy	24 (6.1%)	21 (5.7%)
Gastrointestinal/liver-biliary tract	42 (10.7%)	31 (8.3%)
Head and neck	53 (13.5%)	71 (19.1%)
Inguinal/scrotum	69 (17.5%)	57 (15.3%)
Pediatric Gynaecology	6 (1.5%)	9 (2.4%)
Skin/soft tissue	44 (11.2%)	30 (8.1%)
Umbilical hernia/abdominal wall hernia	75 (19%)	74 (19.9%)
Thoracic	16 (4%)	28 (7.5%)
Urologic	17 (4.2%)	12 (3.2%)
Other	49 (12.3%)	39 (10.5%)

No difference between the different wound classes was reported between the two study populations: clean wounds were 300 (76.1%) in pre- and 301 (80.9%) in post-, clean-contaminated wounds were 63 (16.0%) in pre- and 52 (14.0%) in post- and contaminated wounds were 31 (7.9%) in pre- and 19 (8.4%) in post-.

No significant difference in the type of surgical procedures was reported between the pre- and post-intervention period, as 184/394 (46.7%) and 153/372 (41.1%) patients received a PAP during pre- and post-intervention period respectively (Table 2). In addition, the number of patients receiving PAP according to the guidelines indications increased from 152/184 in pre- (82.6%) to 132/153 in post- (86.3%), even though the difference was not statistically significant (p value 0.4) (Table 2).

**Table 2:** Comparison between PAP administration and correct indication in pre- and post-intervention period

	Pre-intervention (n = 394)	Post-intervention (n = 372)	p-value
Administration of PAP			
Yes	184 (46.7%)	153 (41.1%)	0.12
No	210 (53.3%)	219 (58.9%)	0.12
Correct indication for PAP^a^	n = 184	n = 153	
Yes	152 (82.6%)	132 (86.3%)	0.4
No	32 (17.4%)	21 (13.7%)	0.4

^a^Indication for PAP is calculated only for patients who received PAP (184 in pre-inteverntion and 153 in post-). Indication for PAP is considered correct when it follows the guidelines’ recommendations

In the post-intervention period, there was an increase of correct PAP administration with 90/184 (48.9%) in pre- versus 93/153 (60.0%) in post-intervention period (p = 0.03) (Table 3).

**Table 3:** Correct PAP and most prescribed antibiotics in pre- and post- intervention period

	Pre-intervention (n = 184)	Post-intervention (n = 153)	p-value
Correct PAP^a^			
Yes	90 (48.9%)	93 (60.1%)	0.03
No	94 (51.1%)	60 (39.2%)	0.03
Antibiotic			
Cefazolin	145 (78.8%)	146 (60.1%)	0.0001
Metronidazole	45 (24.5%)	35 (22.9%)	0.99
Amoxicillin/clavulanic acid	35 (19%)	19 (12.4%)	0.36
Ampicillin/sulbactam	37 (20.1%)	9 (5.4%)	0.0003
Other	7 (3.5%)	6 (3.6%)	0.7

^a^PAP is considered correct when administered as recommended by guidelines both in terms of type of antibiotic and duration of administration

In the post-intervention period, there was an increase of cefazolin use from 78.8 to 87.0% (p = 0.0001) with a decrease of ampicillin/sulbactam from 20.1 to 5.4% (p = 0.003) as suggested by the CPs (Table 3).

Indeed, we found that the selection of the appropriate antibiotic for prophylaxis improved in the post-intervention period, both for monotherapy from 81.0 to 91.9% (p = 0.02) and combination therapy from 65.9%) to 100% (p = 0.004) (Table 4).

**Table 4:** Choice of antibiotic for PAP and time of discontinuation of antibiotics after surgical procedure

Choice of antibiotic for PAP^a^	Pre-intervention (n = 184)	Post-intervention (n = 153)	p-value
Monotherapy	n = 137	n = 124	
Correct	111 (81%)	114 (91.9%)	0.02
Not correct	26 (19%)	10 (8.1%)	0.02
Combination therapy	n = 47	n = 29	
Correct	31 (65.9%)	29 (100%)	0.004
Not correct	16 (34%)	0 (0%)	0.004
Discontinuation within 24 h	n = 184	n = 153	
Yes	83 (45.1%)	102 (66.7%)	< 0.001
No	101 (54.9%)	51 (33.3%)	< 0.001

^a^choice of monotherapy versus combination therapy was considered correct according to the guidelines’ recommendations

Moreover, the duration of prophylaxis decreased during the post intervention period, with an increase of PAP discontinuation within 24 h, from 83/202 (45.1%) in the pre-intervention period to 102/153 (66.7%) (< 0.001) (Table 4).

The stratification of the population by type and characteristics of the surgical procedures showed how CP was significantly effective especially for emergency procedures and for all surgical procedures involving head/neck and thorax (Table 5).

**Table 5:** PAP Stratification by wound class and by type of procedure

	Pre-intervention (n = 90)	Post-intervention (n = 93)	*p*-value
Wound class			
Clean (C)	48 (53.3%)	57 (61.2%)	
Clean-Contaminated (CC)	24 (26.6%)	22 (24.4%)	
Contaminated (CO)	18 (20%)	14 (15.4%)	
Dirty-Infected (D)	0 (0%)	0 (0%)	
Emergency procedure			
Yes	8 (8.8%)	21 (22.5%)	0.02
No	82 (91.2%)	71 (77.5%)	0.02
Type of procedure			
Appendectomy	16 (17.7%)	16 (17.2%)	0.9
Gastrointestinal/liver-biliary tract	24 (26.6%)	16 (17.2%)	0.12
Head and neck	2 (2.2%)	10 (10.7%)	0.2
Inguinal / scrotum	10 (11.1%)	5 (5.3%)	0.15
Paediatric Gynaecology	1 (1.1%)	4 (4.3%)	0.18
Skin/soft tissue	11 (12.2%)	9 (9.6%)	0.75
Thoracic	1 (1.1%)	9 (9.6%)	0.03
Urologic	6 (6.6%)	7 (7.5%)	0.95
Other	19 (21.1%)	17 (18.2%)	0.93

SSIs rate assessment showed no difference between the two analyzed periods (10/394 (2.5%) in pre- vs 7/372 (1.9%) in post).

## Discussion

Perioperative antibiotic prophylaxis is the most effective intervention to prevent SSIs [1]. The most recent guidelines [9] define procedures requiring PAP, recommending narrow spectrum antibiotics as first choice for less than 24 h for all procedures (with the exception of cardiac surgery). So far, few studies developed an antimicrobial stewardship program to improve antibiotic prescriptions on PAP in children. Three of these studies showed an improvement of antimicrobial prescriptions after the implementation of perioperative guidelines [3, 6, 7] while Putnam et al. reported no improvement despite multiple interventions [8].

Despite the availability of consensus guidelines designed to facilitate the appropriate use of PAP, a significant variation in this practice has been found for the most commonly performed operations in pediatric surgery [12].

On 31 January 2017 the CP (Figs. 2, 3, and 4) for PAP was implemented and on the same day, an educational lecture was presented. After the lecture, a pocket card was delivered to all participants.

As reported by the studies above mentioned [3, 6, 7], also in our Centre the compliance to PAP guideline improved after CP implementation. Correct PAP significantly increased from 48.9 to 60.1%, with a change both in first choice antibiotics and in duration of prophylaxis.

The choice of correct monotherapy accounted for 81% in pre-intervention period reaching 91.9% after CP implementation. Cefazolin, the most prescribed antibiotic in both periods, definitely became the first choice in post-intervention period with a concomitant decrease of ampicillin/sulbactam. This change affected especially head/neck and thorax procedures, where ampicillin/sulbactam was the drug of choice before the intervention. Indeed, PAP CP recommends cefazolin as the first-line antibiotic for all the procedures due to its activity against S. aureus (MSSA) and Gram-negative bacteria, its narrow-spectrum and its low cost. Ampicillin/sulbactam should be considered an alternative only for its broader spectrum [9].

Moreover, the use of correct combination therapy increased. Again, an important contribution was given by the reduction of ampicillin/sulbactam prescriptions especially in association with metronidazole. Indeed, this combination should be avoided due to their overlapping spectrum of activity against anaerobic bacteria. In the post intervention period, the combination of choice was cefazolin and metronidazole. Also the number of patients with a PAP discontinued within 24 h increased from 83/202 (45.1%) in pre-intervention period to 102 (66.7%) in post-intervention period.

The procedures which have benefitted the most from the intervention were emergency procedures. Usually, patients who undergo emergency surgical evaluation are a severely ill and for this reason surgeons are more prone to exceed the 24 h. Indeed, this represents one of the most difficult points of implementation for an antimicrobial stewardship program. Many are the barriers identified in stopping PAP, the most common being the complexity and duration of surgical procedure, diagnostic uncertainty, inexperienced clinicians, extended in-hospital stay, patient preferences and the fear of SSIs are the most common [3, 13]. The persistence of urinary catheter represents another point of discussion. Even though all the guidelines recommend stopping PAP despite the presence of a urinary catheter, many surgeons are still reluctant. This could be the reason why we have not seen, for urologic procedures, the same improvement we have seen for others. Moreover, many of the current guidelines and specialty-specific recommendations for the pediatric population are based on adult clinical data. It is possible that physicians may not find those guidelines relevant to their pediatric patients. Finally, confusion may exist when indication from adult guidelines are not in line with pediatric observational studies (e.g. inguinal hernia repair) [13].

For a further improvement in PAP compliance rate some authors suggested to enforce guidelines’ effect with and periodic audit by a surgeon trained in antimicrobial stewardship [3]. This physician would monitor the choice, time and dose of PAP administration and would ensure the guidelines adherence.

Moreover, Prado et al. [14] demonstrated how a hospital pharmacist could have a key role, participating in education activities as part of the discussion groups and in managerial actions that optimized the process of ordering, dispensing, administering, and documenting the perioperative antibiotic prophylaxis.

Despite the higher use of narrow-spectrum antibiotic for fewer days, there was no increase in treatment failures between the two analyzed periods.

This study has strengths and limitations. This is the first study that evaluates the effectiveness of antimicrobial stewardship through clinical pathways in an Italian hospital. This intervention was designed to be feasible, generalizable and was developed by a multidisciplinary team to guarantee the best quality and a high level of coordination of interventions.

The primary limitation of our study is the retrospective nature of the analysis. Another limit was the analysis of treatment failure: we collected SSIs information only trough electronic medical records of our centre. Hence, if a patient had been admitted to another one we would miss that information.

## Conclusion

CPs with a proper educational intervention can be a useful tool to improve the choice of first-line antibiotic and the duration of PAP in pediatric patients.
